# Experimental cross species transmission of a major viral pathogen in bees is predominantly from honeybees to bumblebees

**DOI:** 10.1098/rspb.2021.2255

**Published:** 2022-02-23

**Authors:** Anja Tehel, Tabea Streicher, Simon Tragust, Robert J. Paxton

**Affiliations:** ^1^ General Zoology, Institute for Biology, Martin Luther University Halle-Wittenberg, Hoher Weg 8, 06120 Halle (Saale), Germany; ^2^ German Centre for Integrative Biodiversity Research (iDiv) Halle-Jena-Leipzig, Puschstrasse 4, 04103 Leipzig, Germany

**Keywords:** *Apis mellifera*, *Bombus terrestris*, DWV-A, RNA virus, spillover, spillback

## Abstract

Cross-species transmission of a pathogen from a reservoir to a recipient host species, spillover, can have major impacts on biodiversity, domestic species and human health. *Deformed wing virus* (DWV) is a panzootic RNA virus in honeybees that is causal in their elevated colony losses, and several correlative field studies have suggested spillover of DWV from managed honeybees to wild bee species such as bumblebees. Yet unequivocal demonstration of DWV spillover is lacking, while spillback, the transmission of DWV from a recipient back to the reservoir host, is rarely considered. Here, we show in fully crossed laboratory experiments that the transmission of DWV (genotype A) from honeybees to bumblebees occurs readily, yet we neither detected viral transmission from bumblebees to honeybees nor onward transmission from experimentally infected to uninoculated bumblebees. Our results support the potential for viral spillover from honeybees to other bee species in the field when robbing resources from heterospecific nests or when visiting the same flowers. They also underscore the importance of studies on the virulence of DWV in wild bee species so as to evaluate viral impact on individual and population fitness as well as viral adaption to new host species.

## 1. Introduction

Pathogen spillover, the cross-species transmission of a pathogen from a reservoir to a recipient host species, may lead to disease emergence in the recipient host, impacting host community structure and acting as an important cause of biodiversity decline and risk to domestic animal and human health [1–3]. Domesticated animals may represent reservoir hosts and a source of pathogens that spill over into wild species, e.g. *Mycoplasma ovipneumoniae* that spills over from domestic sheep and goats into bighorn sheep (*Ovis canadensis*) [4]. The domestic Western honeybee (*Apis mellifera*), the world's most numerous commercial pollinator [5], may also act as a reservoir host from which pathogens spill over and pose a harm to wild bee species, a worldwide threatened [6] yet economically and ecologically important taxon for their pollination services [7].

*Apis mellifera* is the presumed reservoir host of *Deformed wing virus* (DWV), a (+)ssRNA virus that has become a global emerging infectious disease of honeybees ([8]; reviewed in [9,10]) as a consequence of vector-based transmission by the exotic ectoparasitic mite *Varroa destructor* [11]. Mounting correlation evidence supports the view that DWV spills over from domesticated honeybees into sympatric wild bee species, particularly bumblebees (*Bombus* spp.) [12–15]. Given DWV's high virulence in honeybees [16], the ubiquity of both *A. mellifera* [17] and DWV [8] across terrestrial biomes, and the ongoing decline of wild bee species [6] that may be attributed to pathogen spillover [18], it is important to study DWV's potential for cross-species transmission together with the involved pathways if we are to understand and control its spread and impact.

To explain the first record of DWV in bumblebees, Genersch *et al.* [19] hypothesized cross-species transmission during colony robbing, wherein a bumblebee robs DWV-contaminated honey or hive debris from a collapsing, infected honeybee hive. Supporting this route of horizontal transmission, DWV has been found in a range of other insect species associated with heavily DWV-infected honeybee hives on Hawaii islands [20,21]. Yet *Bombus* spp. and other wild bee species are rarely seen at honeybee hives, arguing against the general importance of this transmission route [22]. For sympatric communities of bee species, shared flowers more likely act as important transmission hubs for a range of bee pathogens [23,24], including viruses such as DWV [22]. In support of this route of transmission, mounting correlational evidence relates DWV prevalence in honeybees to that in bumblebees collected on flowers at the same field sites [12–15].

Yet evidence that flowers act as transmission hubs for the virus is not unequivocal. In support of this hypothesis, Fürst *et al*. [12] found viral sequences of co-occurring honeybees and bumblebees to be identical, suggesting on-going transmission in the field, presumably at flowers. Contrary to this, in a first experiment with the North American *Bombus impatiens* visiting DWV-infected flowers in flight cages, bumblebees failed to acquire DWV [25]. Moreover, in laboratory assays with genetically labelled DWV, Gusachenko *et al.* [26] were able to demonstrate that DWV actively replicates when injected into the Western Palearctic *Bombus terrestris* (see also [27]) but failed to demonstrate viral acquisition and replication by feeding, questioning the spillover of DWV from honeybees to bumblebees through shared resource use at flowers in the field. In a more recent series of flight cage experiments with *B. impatiens* and DWV, Burnham *et al*. [28] have now demonstrated the potential for viral transmission from honeybees to bumblebees as well as transmission from *Bombus* back to *Apis*; DWV-infected honeybees deposited DWV onto red clover (*Trifolium pratense*), *B. impatiens* foraging on DWV-infected flowers became infected with DWV, and DWV-infected *B. impatiens* themselves deposited DWV onto artificial flowers in a laboratory setting. It is unclear whether differences among studies in the potential for transmission of DWV (from *Apis* to *Bombus*) reflect the choice of *Bombus* species or experimental paradigm. Furthermore, there is a need to characterize the onward transmission of DWV from *Bombus* to conspecifics and heterospecifics, including spillback to *Apis*, to understand the epidemiology of DWV and the impact of spillover on host populations [29].

To characterize the potential for, and directionality of, horizontal transmission of DWV between honeybees and bumblebees, we undertook fully crossed laboratory experiments in which we inoculated either the reservoir host *A. mellifera* or the common and widespread *B. terrestris* with DWV derived from honeybees and tested for transmission to uninfected individuals. We provide unequivocal support for transmission of DWV from *Apis mellifera* to *Bombus terrestris* through physical contact and at a shared food resource but detected neither onward transmission of DWV from *B. terrestris* to *B. terrestris* nor transmission back to *A. mellifera*.

## 2. Material and methods

### (a) Experimental set-up

**Figure 1.**
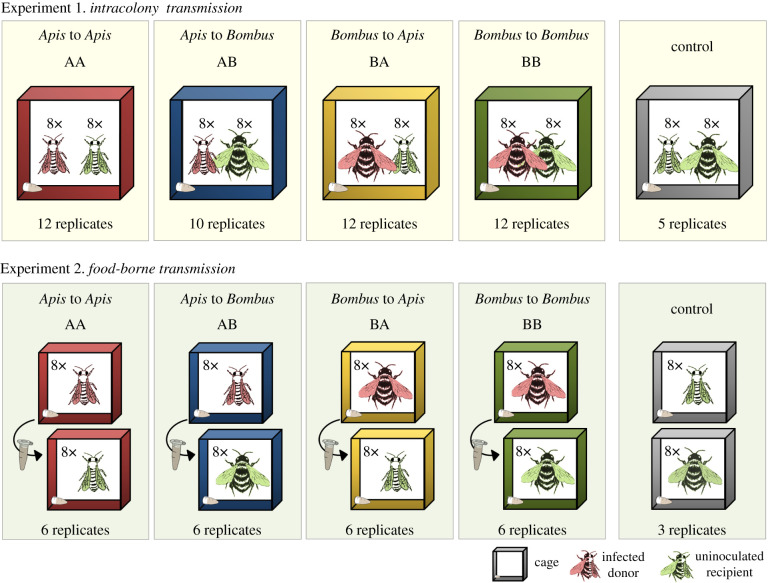
Schematic of the experimental set-up. Viral transmission within and between honeybees and bumblebees was investigated in two experiments in which half of the bees were experimentally inoculated by injection with 10^7^ viral genome equivalents of DWV-A (red, virus donors) while the other half were uninoculated and therefore initially considered uninfected with DWV-A (green, virus recipients). In Experiment 1, mimicking *intracolony transmission*, donor (red) and recipient (green) bees were held together in one cage, permitting multiple horizontal transmission routes. In Experiment 2, mimicking *food-borne transmission*, donor (red) and recipient (green) bees were held in different cages, and every 24 h the feeding tube was transferred from a donor to its paired recipient cage to allow horizontal transmission only via shared food. Both the number of independent replicates (cages per treatment) and bees per cage (8x = eight bees) are given. (Online version in colour.)

We established an experimental paradigm to test for transmission of DWV within and between *A. mellifera* and *B. terrestris* by housing virus-inoculated ‘donor’ bees and uninoculated ‘recipient’ bees in metal cages (10 cm × 10 cm × 6 cm), either mixed in one cage or in single-species cages (figure 1). Additional details on housing bees are given in the electronic supplementary material, methods. For viral quantification, bees where removed from the experimental cages after, respectively, 7 or 14 days, freeze killed and stored at −80°C until further analysis.

#### (i) Experiment 1, mimicking *intracolony transmission*

In the first experiment, donor and recipient bees were mixed in the same cage, and cages held either a single bee species, mimicking intraspecific transmission, or both honeybees and bumblebees, mimicking interspecific transmission. Either honeybees or bumblebees were used as virus donors and as virus recipients in a fully crossed design (figure 1). Though this experiment permitted multiple plausible routes of transmission (faecal–oral, via trophallaxis, shared food or grooming), it maximized bee-to-bee transmission, thereby mimicking the scenario in which bees interact with conspecifics within a hive or when a heterospecific robs the honey stores of the other species.

The donor–recipient combination *Apis* to *Apis* (treatment AA) acted as a positive test of our experimental paradigm as several of the plausible transmission routes within this treatment are well established for DWV among honeybees [22]. We excluded the most important *Apis*–*Apis* vector-based transmission route via the ectoparasitic mite *V. destructor* because it is restricted to *Apis* spp. and is not known to parasitize *Bombus* [22]. We did not detect *V. destructor* in any of our cages.

Under the plausible assumption that *A. mellifera* is the reservoir host of DWV, the combination *Apis* to *Bombus* (treatment AB) mimicked virus spillover. The combination *Bombus* to *Apis* (treatment BA) mimicked potential spillback, though we infected our donor *B. terrestris* experimentally and therefore our protocol did not strictly fulfil the definition of spillback, which is the transmission of a pathogen from reservoir to recipient host species and its subsequent transmission back to the reservoir host. Finally, the treatment *Bombus* to *Bombus* (treatment BB) tested for potential onward viral transmission, e.g. within a *Bombus* colony (figure 1) of a honeybee-derived viral inoculum.

Viral donor bees were generated by briefly placing individual workers on ice and then injecting them laterally between the second and third tergite with 10^7^ viral genome equivalents of DWV genotype A using a Hamilton syringe (hypodermic needle outer diameter: 0.235 mm), sufficient to guarantee infection of all individuals [27]. Donor bees were held for 24 h in a cage (16 bumblebees per cage or 24 honeybees per cage) to ensure they survived physical handling (injection). Surviving bees were anaesthetized with CO_2_ for 3 min to facilitate handling and then eight of them were transferred to a new cage simultaneously with eight anaesthetised but un-injected recipient bees, representing day 1 of the experiment. Donor and recipient bees were labelled by clipping 3 mm off their right or left forewing, respectively.

Each treatment was replicated 10–12 times (electronic supplementary material, table S1). As a negative control, five cages each with eight untreated honeybees and eight untreated bumblebees were established and maintained as described above to check for background infection as a consequence of the experimental paradigm; none was infected (electronic supplementary material, table S1).

Bees (donor and recipient) were analysed at day 7 post-introduction by real-time quantitative PCR (qPCR; see electronic supplementary material, methods) to test for transmission and to quantify DWV titre. To do so, bees were removed from the experimental cages, freeze killed and immediately thereafter stored individually at −80°C until further analysis. Aggressive behaviour between honeybees and bumblebees in heterospecific treatments was frequently observed, potentially leading to a lowered force of infection from donors to recipients. We did not, however, record a difference in viral titre in recipient bees in cages with a high versus low force of infection, measured as the number of donor bees alive at day 5 (electronic supplementary material, figure S1). Survival in single-species cages was high through to day 5, averaging greater than 70% across cages (electronic supplementary material, table S1).

#### (ii) Experiment 2, mimicking *food-borne transmission*

In the second experiment, mimicking food-borne transmission via a common food source, donor and recipient bees were established as described for Experiment 1 but maintained in separate cages throughout. Cage establishment represented day 1 of the experiment. To mimic transmission (faecal−oral and oral−oral) at e.g. flowers, a donor cage's feeding tube was transferred every 24 h to its paired recipient cage while donor cages received a new feeding tube. Both honeybees and bumblebees were used as virus donors and as virus recipients in a fully crossed experimental design comprising six pairs of cages per treatment (figure 1; electronic supplementary material, table S2), with the treatment *Apis* to *Apis* (AA) acting as a positive control [22], *Apis* to *Bombus* (treatment AB) mimicking spillover, *Bombus* to *Apis* (treatment BA) the potential for spillback and *Bombus* to *Bombus* (treatment BB) onward transmission to other *Bombus* individuals.

Donor and recipient bees were tested by qPCR for DWV titre at days 7 and 14 post-introduction as a measure of transmission (sample sizes in electronic supplementary material, table S2); removed bees were immediately frozen and stored individually at −80°C until analysis. To hold constant the force of infection throughout the experiment, bees that died or were removed for viral quantification from donor cages were replaced with additional, experimentally infected conspecifics of the donor. As in Experiment 1, we did not detect *V. destructor* in any of our cages.

Additionally, we established three cages per bee species with either eight untreated honeybees or eight untreated bumblebees and maintained them with their unique feeding tube. As for Experiment 1, these ‘negative control’ cages were checked for background infection as a consequence of the experimental paradigm.

### (b) Source of bees

*Bombus terrestris* is a dominant bee species in temperate European ecosystems that harbours several honeybee-associated viruses in the wild, including DWV (e.g. [12–24]). We used commercial *B. terrestris* colonies that were fed UV-radiated, freshly defrosted pollen pellets and honeybee colonies originating from our institute apiary in Halle (Germany) and originally purchased from local beekeepers. Prior to use, all colonies and pollen were tested by real-time qPCR for the presence of six common honeybee viruses, including DWV (electronic supplementary material, table S3). Bees from different colonies were evenly distributed between experiments and treatments (donor, recipient and control) to exclude potential genetic effects. Age of bees was not controlled as we deemed it unnecessary; our experimental endpoint was viral titre and not survival or behaviour. Additional details on the source of bees as well as the number of colonies and individuals used in experiments are given in the electronic supplementary material, Methods.

### (c) Viral inoculum

Two widespread genotypes of DWV, namely A and B, can be found in co-occurring honeybees and bumblebees [12]. We chose to use DWV genotype A (DWV-A) in experiments because our previous study suggested that it (but not DWV genotype B) compromised bumblebee survival when stressed by starvation [27]. Our DWV inoculum was the same as that of Tehel *et al.* [27]. In short, we propagated DWV-A, originally derived from a honeybee, in honeybee pupae using methods described in Tehel *et al.* [30]. Absolute viral quantification was by qPCR. Ultradeep next-generation sequencing on an Illumina platform confirmed the identity of our DWV-A inoculum and the absence of other pathogens (see [30] and BioProject ID PRJNA515220).

### (d) Viral quantification

Absolute quantification of viral titre by qPCR was performed on individual bees throughout. It followed previously described methods ([27,30]; see electronic supplementary material for RNA extraction, cDNA synthesis and qPCR protocols and quality checks, which included technical duplication of qPCRs, the inclusion of positive and negative controls on each qPCR plate and a qPCR quantification cycle (Cq) threshold of Cq < 35 (approximately equivalent to 10^6^ genome equivalents per bee)) to define a positive sample. Sample sizes of bees analysed for DWV are given in the electronic supplementary material, tables S1 and S2 (Experiments 1 and 2, respectively) and viral titres are presented in the text as mean genome equivalents per bee ± s.e.m. Samples used for laboratory analysis were randomly selected from all frozen bees when available for a given treatment.

### (e) Statistics

All analyses were performed in R v. 3.5.1 (R Core Team).

We used Fisher exact tests (package ‘stats') to compare proportions of infected versus non-infected bees. In Experiment 1, analyses were based on individual bees, assuming independence of individuals in a cage (results were qualitatively similar when analysing data at the level of the cage). In Experiment 2, transmission could only have occurred between cages via the shared source of food but, once one individual within a recipient cage became infected, transmission within a cage could have subsequently occurred via additional routes; for Experiment 2, cage was therefore used as the statistical unit of replication.

To assess differences in log_10_-transformed viral titres, we used two different models for the donor–recipient combinations in Experiment 1: one for viral titres with *Apis* as a donor (treatment AA and AB with four levels: donor in AA, recipient in AA, donor in AB and recipient in AB) and one with *Bombus* as a donor (treatment BA and BB with only two levels: donor in BA and donor in BB, as the recipients never became infected). We used a linear mixed model (LMM, package ‘lme4’ [31]), with experimental cage as a random factor for the donor–recipient combinations with *Apis* as a donor to account for the fact that we measured two bees from the same cage for donors in treatment AA and receivers in treatment AB in one out of five and two out of seven cages, respectively. A linear model (LM) was used to analyse donor–recipient combinations with *Bombus* as a donor because only one donor was analysed per cage.

In Experiment 2, as in Experiment 1, we used two different models to analyse viral titre data for the donor–recipient combinations, an LMM for viral titres with *Apis* as a donor (treatment AA and AB with two levels: donor in AA and donor in AB, as only very few recipients became infected) and an LM for viral titres with *Bombus* as a donor (treatment BA and BB with only two levels: donor in BA and donor in BB, as the recipients never became infected). An LMM with experimental cage as a random factor was again used for the donor–recipient combinations with *Apis* as a donor to account for the fact that we measured two bees from the same cage for donors in treatment AA in one out of five cages. An LM was instead used to analyse donor–recipient combinations with *Bombus* as a donor as we only analysed one donor per cage. For all analyses, models were compared to null (intercept only) models to assess whether levels of donor–recipient combinations in the respective models were significant predictors of viral titre.

Pairwise comparisons between factor levels of a significant predictor were performed using *post hoc* tests, adjusting the family-wise error rate according to the method of Westfall (package ‘multcomp’ [32]). Model assumptions were checked with diagnostic tests and plots implemented in the package ‘DHARMa’ [33] for LMMs, or via diagnostic plots, the Shapiro–Wilks Test and the Bartlett-Test in base R for LMs.

## 3. Results

### (a) Experiment 1, mimicking intracolony transmission

**Figure 2.**
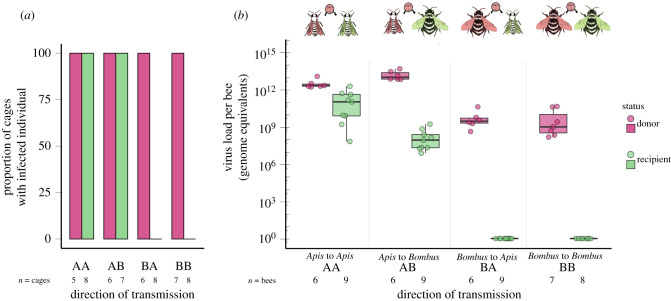
Experiment 1, mimicking intracolony transmission. (*a*) The proportion of cages in which experimentally infected donor bees (red, 1st column of a treatment) and uninoculated recipient bees (green, second column of a treatment) were infected with DWV by day 7. (*b*) Viral (DWV) titres of donor and recipient bees at day 7. Donors (red, injected with 10^7^ viral genome equivalents of DWV-A) and recipients (green, DWV-uninoculated) shared one cage, permitting multiple horizontal transmission routes and mimicking elements of intracolony transmission. All donor bees had high viral titres in all cages by day 7. All recipient *Bombus* from the treatment AB showed high titres by day 7, indicative of infection, whereas none of the recipients from the transmission treatments in which *Bombus* was the donor (BA and BB) was infected. Sample sizes given as (*a*) number of cages and (*b*) number of analysed bees. (Online version in colour.)

In Experiment 1, we could clearly demonstrate viral transmission from infected honeybees to uninoculated recipient bumblebees. All nine recipient bumblebees from the subset we analysed (treatment AB: nine of nine individuals) were infected with DWV-A after 7 days of contact with infected donor honeybees (figure 2*a*), whereas none of the five analysed bumblebees was infected in a control cage (control: five of five *Bombus* individuals analysed; comparison of infection status of treatment versus control, Fisher exact test *p* < 0.001; electronic supplementary material, table S1). All donor bees were successfully infected (figure 2*a*). These data demonstrate that infected honeybees readily transmit virus to the bumblebee *B. terrestris* when in close contact.

In the treatment AA, all nine analysed recipient honeybees (nine of nine individuals) were infected by donor honeybees (figure 2*a*), whereas no analysed honeybee was infected in a control cage (control: five of five *Apis* individuals; comparison of infection status of treatment versus control, Fisher exact test *p* < 0.001; electronic supplementary material, table S1 and figure S2A), confirming *Apis*–*Apis* transmission and that our experimental paradigm functioned as expected.

In the two treatments AB and AA in which honeybees were viral donors, viral titres in all donor honeybees and all recipient bees were consistently high (figure 2*b*), often orders of magnitude greater than the inoculum (10^7^) injected into donors (mean ± s.e.; donor honeybees of both treatments: 1.19^13^ ± 4.27^12^, *n* = 12; recipient *Bombus* in treatment AB: 3.44^8^ ± 5.86^8^; recipient *Apis* in treatment AA: 3.63^11^ ± 2.16^11^, *n* = 9 each), evidence for transmission from donor to recipient and replication within recipients. Additional statistical results comparing viral titres across groups are found in the electronic supplementary material, Results.

In stark contrast with our results in which honeybees were viral donors, we found no evidence of viral transmission from donor bumblebees, either to recipient honeybees or to recipient bumblebees in treatments BA and BB, respectively. All nine analysed recipient honeybees (treatment BA: nine of nine individuals) and all eight analysed recipient bumblebees (treatment BB: eight of eight individuals) were devoid of DWV-A after 7 days of contact with infected donor bumblebees (figure 2*a*; electronic supplementary material, table S1). All donor bumblebees were infected (treatment BA: six of six donor individuals in six of six cages, Fisher exact test of difference in infection status between donor and recipient, *p* < 0.001; treatment BB: seven of seven donor individuals in seven of seven cages, Fisher exact test of difference between donor and recipient, *p* < 0.001; figure 2*a*; electronic supplementary material, table S1A). Bumblebees in control cages remained uninfected with DWV-A (control: five of five *Bombus* individuals; electronic supplementary material, table S1B and figure S2A), confirming that our experimental paradigm to introduce DWV-A infected donors and DWV-A uninfected recipients into cages had functioned.

Viral titres in all donor bumblebees were consistently high (mean ± s.e.; donor bumblebees of both treatments: 1.19^10^ ± 5.25^9^, *n* = 13), indicating successful viral infection of donor bumblebees (figure 2*b*). Additional statistical results comparing viral titres across groups are given in the electronic supplementary material, Results.

### (b) Experiment 2, mimicking food-borne transmission

**Figure 3.**
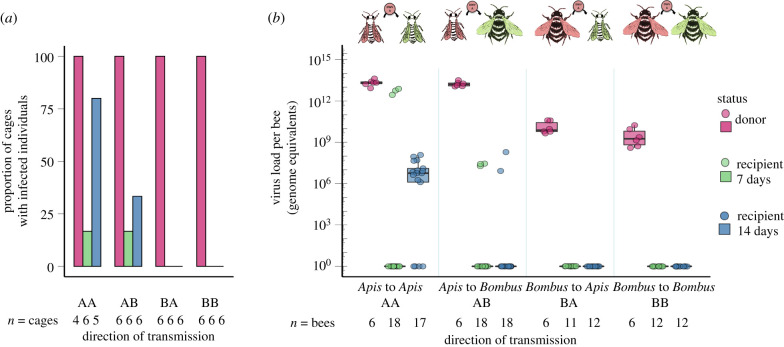
Experiment 2, mimicking food-borne transmission. (*a*) The proportion of cages in which experimentally infected donor bees (red, 1st column of a treatment) and uninoculated recipient bees (second and thirrd columns of a treatment) were infected with DWV by day 7 (second column: green) and day 14 (third column: blue). (*b*) Viral (DWV) titres of experimentally infected donor bees at day 7 and of recipient bees at days 7 (green) and 14 (blue). Donors (red, injected with 10^7^ viral genome equivalents of DWV-A) and recipients (green and blue, DWV-uninoculated) were held in separate cages but the donor feeding tube was moved from a donor to its paired recipient cage every day, mimicking the use of a shared food resource. All donor bees showed high viral titres at day 7. Recipient bees from treatments in which honeybees were the donors (*Apis* to *Apis* and *Apis* to *Bombus*) showed high titres at days 7 and 14, indicative of infection, whereas recipient bees from treatments in which bumblebees were the donors (*Bombus* to *Apis* and *Bombus* to *Bombus*) did not. Sample sizes given as (*a*) number of cages and (*b*) number of analysed bees. (Online version in colour.)

In Experiment 2, we could again demonstrate viral transmission from donor honeybees to recipient bumblebees and to recipient honeybees, though with reduced efficiency compared to Experiment 1. At day 7, recipient *Bombus* and recipient *Apis* in one of six cages apiece were infected with DWV (treatments AB and AA, respectively; figure 3*a*; electronic supplementary material, table S2A). This is a significantly lower probability of transmission than in Experiment 1 (Fisher exact test *p* = 0.002 for both treatment AB and for treatment AA). By day 14, two of six and four of five recipient cages contained infected *Bombus* and *Apis* in treatments AB and AA, respectively (figure 3*a*; electronic supplementary material, table S2A), demonstrating that, with time, sharing of food resources leads to successful viral transmission from honeybees to bumblebees (and to conspecific honeybees). Summing across both days 7 and 14, recipient bees became infected in a total of 8 of 12 cages with *Apis* as donors (the cage-wise transmission from *Apis* donors to recipients (honeybees and bumblebees) was 0.67).

Recipient honeybee cages in the treatment AA were either all infected or all non-infected at either of the two time points of sampling (*n* = 6 cages over two time points; electronic supplementary material, figure S3 and table S2A). By contrast, infected recipient bumblebees in the treatment AB were singletons in two of three cages housing infected recipients (electronic supplementary material, figure S3 and table S2S). These data suggest that, if a *B. terrestris* becomes infected through consumption of virus-laden food, there is a low probability that the virus is transmitted onwards to other conspecific bumblebees.

Few recipient bumblebees in treatment AB were infected at day 7 (*n* = 3 of 18 bees) or at day 14 (*n* = 2 of 18 bees; electronic supplementary material, table S2A). Yet the substantive DWV titres of infected recipient bumblebees (mean ± s.e.: 5.53^7^ ± 3.49^7^, *n* = 5; figure 3*b*) are support for viral transmission to and subsequent replication within recipient bumblebees. Additional statistical details comparing viral titres between groups are given in the electronic supplementary material, Results.

Viral titres in donor honeybees inoculated with 10^7^ genome equivalents of DWV were consistently high at day 7 (*Apis* donors in treatment AA: 2.03^13^ ± 2.76^12^, *n* = 12; figure 3*b*), demonstrating successful viral replication in donors. Few recipient honeybees in treatment AA were infected at day 7 (*n* = 3 of 18 bees) but many more were infected by day 14 (*n* = 13 of 17 bees; electronic supplementary material, table S2A). Viral titres of infected recipient honeybees were also greater than 10^7^ (figure 3*b*), supporting horizontal transmission to conspecific honeybees of virus in shared food and subsequent replication of the virus in recipients. Additional statistical details comparing viral titres between groups are given in the electronic supplementary material, Results.

We found no evidence for viral transmission from donor bumblebees to recipient bees, either uninoculated bumblebees or uninoculated honeybees, suggesting that virus is not transmitted onward from one infected *Bombus* to another or transmitted back to *Apis* (figure 3*a*; electronic supplementary material, table S2A). Cages with *Apis* as donors led to far higher transmission to recipient bees than cages with *Bombus* as donors (Fisher exact text *p* = 0.001, odds ratio infinity, 95% confidence intervals 3.055 – infinity). To account for the low number of cages per treatment, we use the binomial theorem to state with 95% confidence that the cage-wise transmission from *Bombus* donors to recipients (honeybees and bumblebees) was less than 0.22.

Inoculated donor bumblebees had high viral titres (1.13^10^ ± 4.02^9^, *n* = 12; figure 3*b*), demonstrating their competence as hosts of DWV. Furthermore, bumblebees were observed to sit on feeding tubes, which were spotted with excretions, suggesting that feeding tubes offered a plausible route of food-borne transmission to recipient bees. Additional statistical details comparing viral titres between groups are given in the electronic supplementary material, Results.

Of the control treatments in Experiment 2 (*Bombus n* = 6, *Apis n* = 6), one bumblebee had a background infection on day 14 of 3.79^7^ genome equivalents, probably because its source colony carried a low-titre infection (electronic supplementary material, figure S2B). We could not detect virus in any other controls.

## 4. Discussion

Our experiments demonstrate that infected honeybees readily transmit DWV-A to the bumblebee *B. terrestris*, both when in close contact and indirectly, when sharing a common food resource (sugar solution). But we could not detect viral transmission from *B. terrestris* to conspecifics or to honeybees, either through direct contact or indirectly via shared food. Our data support the view that DWV-infected honeybees readily transmit virus to *B. terrestris,* which causes an infection, but DWV-infected bumblebees are far less likely to transmit virus back to honeybees or onward to other *B. terrestris*.

Previous studies have been contradictory, arguing either that DWV is unlikely (for *B. terrestris* [26]) or is likely (for *B. impatiens* [28]) to be transmitted from honeybees to bumblebees. Differences among studies might be due to variation among recipient host species in their competence for viral replication or in the mode of transmission used in experiments. *Bombus terrestris* is a susceptible host for DWV when inoculated by injection or by feeding [26,27]. We now show that *B. terrestris* also readily becomes infected when housed with, or when sharing a common source of food with, infected *A. mellifera* under our experimental conditions.

We found that viral spillover from honeybees to bumblebees was more efficient when insects were in direct contact (our Experiment 1 mimicking intracolony transmission) than through a shared food resource (our Experiment 2 mimicking food-borne transmission). Both experiments permitted multiple modes of transmission: faecal−oral and oral−oral for both experiments, as well as via grooming and trophallaxis for Experiment 1 mimicking intracolony transmission. Though vector-based transmission could lead to more efficient transmission within colonies in nature, we did not detect any mites in our cages that could lead to viral transmission and therefore assume that this route does not explain our results. Furthermore, honeybees and bumblebees generally host their own mite species [34,35], additionally arguing against a role for viral vectors in explaining the efficiency of transmission when heterospecific hosts were in direct contact. We hypothesize that interspecific and intraspecific (honeybee to bumblebee, honeybee to honeybee) transmission was likely more efficient in Experiment 1 because it permitted a higher dose of infective virus to be transferred from donor to recipient. Dose is considered critical for cross-species transmission in other cases of viral spillover e.g. MERS-CoV and Nipah virus [36]. In support of our hypothesis, the first detection of DWV in bumblebees was in *Bombus* bearing deformed wings and collected from a honeybee apiary, suggesting that spillover was facilitated by intracolony transmission following bumblebee entry into infected honeybee colonies [19].

That infected bumblebees in our experiment did not lead to the transmission of DWV back to honeybees or onward to uninoculated bumblebees suggests that infected *B. terrestris* are incapable of shedding infective DWV or of shedding sufficient virions to represent an infective dose for a recipient host. In a cage experiment, Burnham *et al.* [28] have shown that *B. impatiens* inoculated with 3 × 10^6^ DWV *per os* subsequently deposit detectable virus on artificial flowers, demonstrating the potential for infected bumblebees to transmit DWV. However, there is no information on the viability and the infectious potential of these shed viruses. Donor *B. terrestris* in our experiments had high viral titres (greater than 10^9^) but had been inoculated by injection. Differences between studies may therefore reflect variation among *Bombus* species in response to viral infection or mode of inoculation. Furthermore, the origin of the inoculum might also determine its transmissibility. The source of our DWV-A was an infected honeybee, and we amplified it in honeybee pupae to generate our experimental inoculum. DWV-A derived from bumblebees might be more transmissible from bumblebee hosts to recipient conspecifics and heterospecifics. Additional analyses of the infectivity of the viruses in oral and anal excretions of bumblebees infected orally or by injection with *Bombus*-derived versus *Apis*-derived inocula would help to resolve these questions.

Shared food resources such as bird feeders or waterholes are a common site of pathogen transmission [37,38]. For bees and other flower-visiting insects, flowers are considered important transmission hubs for their pathogens [23], and observational and experimental data support their role in the transmission of numerous eukaryote pathogens [39–44]. Their role in viral transmission is less well documented; flower-based transmission may theoretically represent a barrier to transmission as many viruses are sensitive to UV light [45,46], and flowers represent an alien and potentially hostile environment for viruses [23]. DWV in particular is considered unstable outside of its host [47]. However, pollen collected from honeybee-visited flowers has been shown to contain infective DWV [48]. Furthermore, DWV is excreted in the faeces of infected honeybees [49], and faeces are deposited on flowers by bees when foraging (for *Bombus*: [50]). Our results in Experiment 2 mimicking food-borne transmission also support the view that DWV is readily transmitted by honeybees to conspecifics and heterospecifics at flowers, either oral−faecally (via faeces) or oral−orally (via cephalic secretions or regurgitation). Floral transmission may well account for the presence of DWV in a wide diversity of flower-visiting insects [51]. We note, however, that our experimental paradigm may well have accentuated food-borne transmission beyond that which may occur naturally at flowers because donors had 24 h to walk over, defecate upon and regurgitate onto a feeding tube whereas flowers are usually visited briefly by foraging bees. Whether *Bombus* spp. transmit DWV (or other viruses) at flowers in the field remains an open question, though an important one to answer to understand the epidemiology of DWV in pollinator communities.

Pathogen spillover *sensu stricto* has been conceptually represented as a series of hierarchical steps, from the release of viable, transmissible environmental stages (virions in the case of viruses like DWV) from a reservoir host species through to successful acquisition by a recipient host species [52,53]. Successful replication in and subsequent transmission among recipient host individuals are additional bottleneck steps that, if overcome by a pathogen, may then lead to disease emergence [29]. We have here shown that DWV-A has the potential to spill over from *A. mellifera* to *B. terrestris*, though we have no support for its onward transmission among recipient bumblebees. That bumblebees, other wild bee species and many other flower-visiting insects often harbour DWV [51], sometimes to titres as high as in honeybees [54], demonstrates the potential for DWV to spill over into other host species and replicate in them. The correlation across field sites in the prevalence of DWV in honeybees and bumblebees [12,14,15] as well as the sequence identity of viral variants in *Apis* and *Bombus* from the same site [12,14,55,56] support the notion of pervasive, ongoing spillover. Given the considerable evolutionary potential of RNA viruses [57], there is a tangible risk of local adaptation of DWV to a bumblebee or other flower-visiting host, with negative knock-on effects on biodiversity and the ecosystem service of pollination.

There is mounting evidence for the impact of pathogens on pollinator species [18]; transcriptome analysis of the rare and declining *Bombus terricola* of North America points to pathogen (and pesticide) driven decline [58]. In our experiments, we employed commercially sourced *B. terrestris*, a common and widespread species [59,60] that may have been inadvertently selected for tolerance to or limited transmission of DWV in breeding facilities. Given the ubiquity of DWV in terrestrial biomes worldwide, its role in driving population loss of rare or declining species needs to be taken seriously.

## Data accessibility

All data supporting the findings of this study are available from the Dryad Digital Repository: https://doi.org/10.5061/dryad.w0vt4b8t7 [61]. The data are provided in the electronic supplementary material [62].
